# From transcription to export: mRNA’s winding path to the cytoplasm

**DOI:** 10.1016/j.tibs.2025.06.004

**Published:** 2025-07-15

**Authors:** Murray Stewart

**Affiliations:** 1 Medical Research Council (MRC) Laboratory of Molecular Biology, Francis Crick Avenue, Cambridge Biomedical Campus, Cambridge CB2 0QH, UK

## Abstract

In eukaryotes, the separation of transcription from translation enables extensive mRNA processing (capping, splicing, and polyadenylation) before translation. This review focuses on recent work that provides considerable insight into how mRNAs navigate these processes in which a spectrum of RNA-binding proteins (RBPs) coordinate different processing steps and couple them to nuclear export. Although the principal components in these pathways have been identified, the precise way in which RBPs bind to mRNAs, some aspects of how their binding and release are mediated by DEAD-box ATPases, and the complete structures of some messenger ribonucleoprotein complexes (mRNPs) remain unclear. Moreover, the checkpoints that recognize both completion of mRNA processing and the generation of mature mRNPs, as well as how they are coordinated, are only partially characterized.

## Nuclear mRNA processing pathway

**Figure 1:**
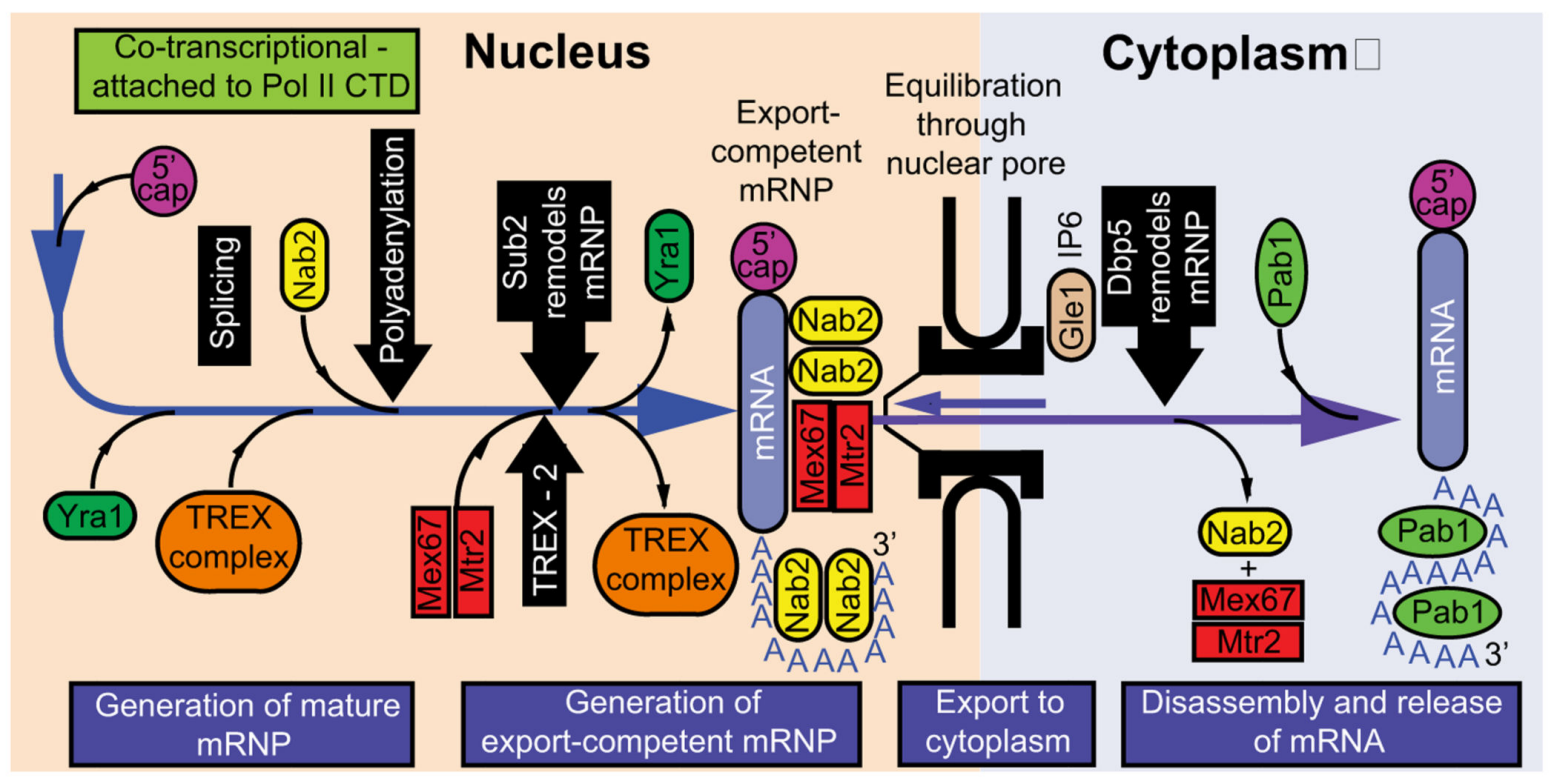
Schematic illustration of the mRNA export pathway. This can be subdivided into four stages: (i) generation of mature messenger ribonucleoproteins (mRNPs) involving 5′ capping, 3′ polyadenylation, splicing, and with the addition of Yra1, the TREX complex, SR proteins, and Nab2. Although these modifications mostly take place co-transcriptionally, some also take place subsequently. (ii) Generation of an export-competent mRNP in which Yra1 and the TREX complex are removed and the Mex67-Mtr2 nuclear export factor is attached. (iii) Export to the cytoplasm though the NPC transport channel facilitated by the interaction between Mex67–Mtr2 and the FG-nucleoporins that overcomes their barrier function. Finally, (iv) disassembly of the mRNP at the NPC cytoplasmic face mediated by Dbp5, Gel1, and IP6 removes Mex67–Mtr2 and Nab2, and releases the transcript into the cytoplasm for translation and prevents its return to the nucleus. Figure modified from [2]. Abbreviations: CTD, C-terminal domain; IP6, inositol hexakisphosphate; NPC, nuclear pore complex; SR proteins, Ser/Arg-rich proteins; TREX complex, transcription-export complex.

The separation of the nuclear and cytoplasmic compartments by the nuclear envelope separates transcription from translation in eukaryotes, and enables mRNA processing (chemical modification) by adding a 5′ cap and a poly(A) tail, as well as removal of any introns, before it is translated. The export of mRNAs though nuclear pore complexes (NPCs) is the culmination of a series of processing and mRNP formation/assembly steps that take place during or immediately following transcription and which serve to ensure that only completely processed mRNAs are delivered to the cytoplasm [1,2]. This pathway can be divided into four major steps, as illustrated in Figure 1, and each step is a separate focus of this review.

Briefly, in the first step, precursor-RNAs (pre-RNAs) generated by the transcriptional machinery are bound by RNA-binding proteins (RBPs) to form mRNPs; the dynamic exchange of specific RBPs within these complexes directs both their subcellular trafficking and interactions with the nuclear processing machinery. This step generates mature mRNAs in which 5′ caps and 3′ poly(A) tails have been added, any introns have been removed, and the mRNP has been detached from the transcriptional, splicing, and polyadenylation machinery. In the second step, mature mRNPs are then remodelled into export-competent mRNPs by removal of many of the RBPs attached during the first step, together with the addition of nuclear export factors. In the third step, nuclear export is mediated by the backwards and forwards equilibration of the export-competent mRNP through the NPC transport channel (Box 1), followed by its disassembly at the cytoplasmic face of the NPC, removing the transport factors, and thereby preventing return of the mRNA to the nucleus and releasing it into the cytoplasm (fourth step).

Although the nuclear mRNA processing, mRNP formation/assembly, and export pathways are similar between different organisms, the pathways in *Saccharomyces cerevisiae* are somewhat simpler and also often more easily investigated than those in metazoans. However, a major difference derives from the presence of the large exon-junction complex (EJC); see Glossary) that is attached following the completion of splicing in metazoans [3–5], but which has no counterpart in S. *cerevisiae*. Throughout this review the corresponding *S. cerevisiae* and metazoan proteins will be referred in the format Yeast/METAZOAN.

Errors or defects in the generation of mature mRNAs or in the nuclear export of mRNPs often impair cells, and these processes are therefore tightly coordinated to inhibit aberrant mRNAs reaching the cytoplasm for translation into proteins. Although considerable information is available about the RBPs and how they interact with one another, some aspects of the way in which they interact with mRNA, and how the conformation of the mRNA (Box 2) may be involved, are less clear. Detailed genetic analysis of the pathway is often complicated because many of the RBPs involved can play multiple roles in mRNP metabolism: for example, in addition to its function as part of the transcription-export (TREX) complex, a multi-protein complex that couples mRNA transcription to its nuclear export [6], UAP56 (an ATP-dependent DEAD-box helicase), is also one of the many components that contributes to splicing [7] and also to resolving co-transcriptionally formed R-loops (hybrid DNA–RNA structures) [7].

### Step 1: the generation of mature mRNPs

The pre-mRNA processing steps that generate a mature mRNA involve 5′ capping, splicing, and 3′ polyadenylation. As it is transcribed, the pre-mRNA becomes compacted by the generation of secondary and higher structures, augmented by a spectrum of RBPs being added as the mRNAs are synthesized [8–11]. Compaction of the pre-mRNA is thought to inhibit its chain becoming entangled with other mRNAs or forming R-loops, as well as affording protection from nucleases [8]). The RBPs also interact with one another, facilitating loading onto the pre-RNA. Because the pre-mRNA is attached to either the transcription, splicing, or cleavage/polyadenylation machinery, mRNPs can generally only be isolated from cells after mature mRNPs have been generated, and this has made it difficult to define the precise ways and timecourses in which different proteins are added.

During transcription initiation, an inverted 7-methylguanosine cap is synthesized to protect the 5′ end from exonucleolytic degradation, promote proper splicing and nuclear export, and facilitate efficient translation initiation. The 5′ cap is immediately recognized and bound by the cap-binding complex (CBP, that contains Cbp80 and Cbp20 in *S. cerevisiae)* that facilitates subsequent processing steps (reviewed in [12]). Although less frequent than in higher eukaryotes, some yeast pre-mRNAs also contain introns that are excised during splicing. The splicing process is mediated by the spliceosome, a large ribonucleoprotein complex (reviewed in [5]). Next, the mature mRNA undergoes 3′ end cleavage and polyadenylation, releasing it from the transcription machinery [2,13]. Nab2 is the primary regulator of poly(A) tail length in *S. cerevisiae* (reviewed in [14]), whereas in mammalian cells an important accessory factor is nuclear PABPN1 [15,16].

Although comprehensive inventories have been obtained of the proteins bound to mature mRNPs, it has been difficult to establish details of the way in which some of these components become incorporated. In addition to proteins bound to the 5′ (CBP 20:80 complex) and 3′ (Nab2 in *S. cerevisiae*, PABNP1 in metazoans) ends, major constituents of mature mRNPs are the TREX complex and **SR proteins**, as well as, in metazoans, the EJC. The *S. cerevisiae* TREX complex is based on a THO complex core to which Yra1/ALYREF, the DEAD-box ATPase Sub2/UAP56 (DDX39B), and Gbp2 and Hrb1 are attached [17,18], whereas SR proteins are characterized by having sequences rich in Ser and Arg (reviewed in [19]). These proteins are thought to become incorporated cotranscriptionally and there often appears to be a level of cooperation between them. In higher eukaryotes, the EJC is also found close to splice sites [3,4] and facilitates TREX recruitment. In long mRNAs in metazoans, the NXF1 transport factor is preferentially required for export of single- or few-exon mRNAs with long exons or high A/U content, whereas TREX complex components preferentially affect spliced and G/C-rich mRNAs [20].

#### TREX complex

The TREX complex is conserved between yeast and metazoans, and contributes to the integration of the nuclear steps of the gene expression pathway and nuclear export [17,18]. The *S. cerevisiae* TREX complex is primarily associated with the transcription machinery, whereas in humans it instead appears to associate primarily with the splicing machinery through its binding to the EJC that is deposited 20 nt upstream of the most 5′ exon–exon junction [5]. At a later stage, the TREX complex contributes to the generation of export-competent mRNPs, although this process appears to be more complex in higher eukaryotes and may also involve relief of autoinhibition based on the arginine-rich N-terminal of NXF1 together with additional components of the TREX complex [21,22].

The TREX complex contains Yra1/ALYREF, the THO complex, and the DEAD-box RNA-dependent ATPase Sub2/UAP56 [17] that can interact with THO [23–25] and with Yra1/ALYREF [26,27]. THO, Sub2/UAP56, and Yra1/ALYREF are evolutionarily conserved from lower to higher eukaryotes [28]. In *S. cerevisiae*, THO recruits Sub2 and Yra1 as mRNAs elongate, facilitating the formation and export of stable mRNPs [29–31], although additional Yra1 appears to bind independently of the TREX complex [32,33]. In human cells, ALYREF is recruited to the cap-binding complex and, in intron-containing mRNAs, by the splicing process [22,34,35], although ALYREF also associates with intronless mRNAs [36] and can, for example, bind to PABPN1 [37]. For both types of mRNA, ALYREF deposition requires the ATPase activity of UAP56 [36,38]. However, the detailed molecular mechanisms by which THO, Sub2/UPA56, and Yra1/ALYREF contribute to the formation of mature mRNPs remain unclear. Moreover, the large number of different mRNAs that are being produced at any one time has frustrated definition of the makeup of the particle produced by a specific mRNA or indeed even knowing whether all mRNPs formed by a specific mRNA are identical.

The structure and interactions between the TREX components have been studied in complexes generated *in vitro* from expressed components [24,25,39], as well as in endogenous complexes [40] and mature mRNPs [32] isolated from *S. cerevisiae*. These studies have established a picture of the S. *cerevisiae* TREX complex in which a dimer formed by the THO complex core acts as a scaffold to which Yra1, Sub2, and other components bind. Human TREX has an analogous structure based on a THO tetramer [23].

##### Yra1/ALYREF

**Figure 2:**
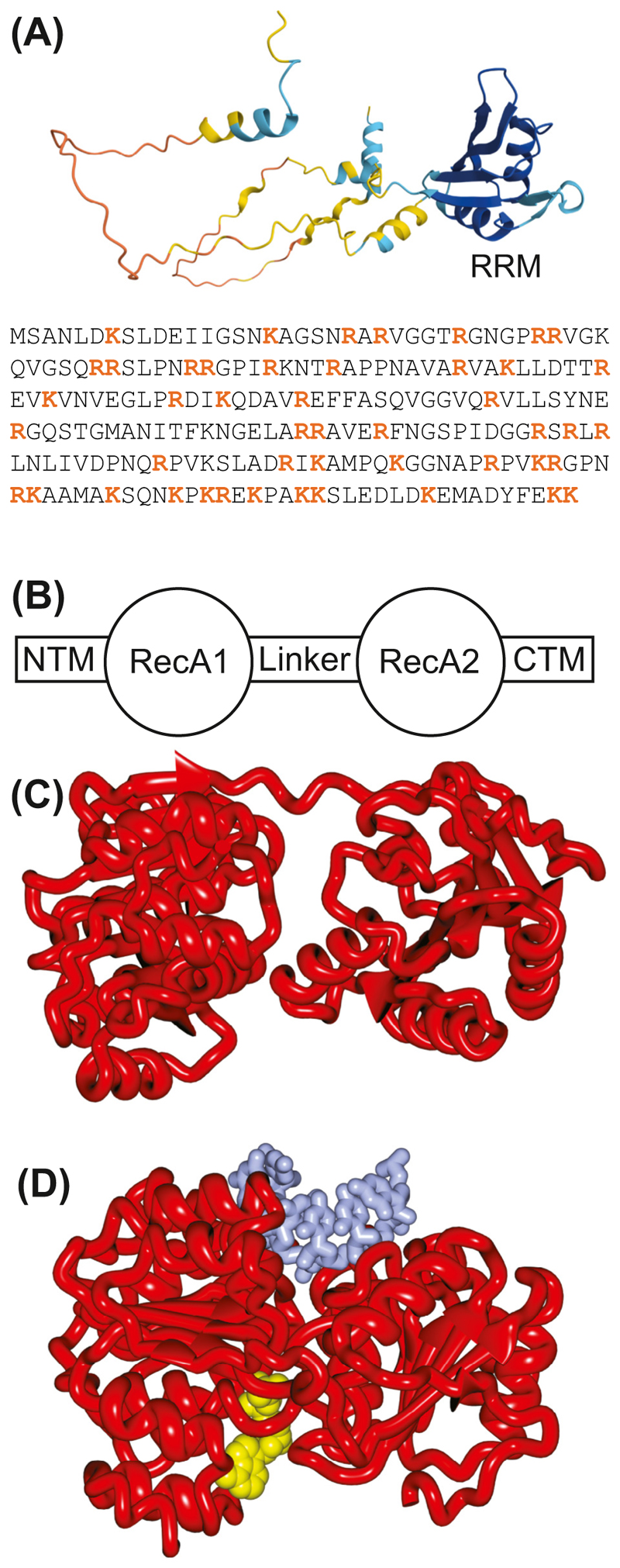
Factors that bind to transcripts to generate mature mRNPs. (A) Structure and AlphaFold representation of Yra1, that has an RNA recognition motif (RRM) together with long unstructured regions that are rich in positively-charged residues (red). (B) Schematic illustration of the DEAD-box ATPase Sub2/UAP56 (Dbp5/DDX19B is similar). (C) The open and (D) closed conformations of Sub2 (based on PDB 1XTI and 5SUP). Abbreviations: CTM, C-terminal region; NTM, N-terminal region.

Yra1/ALYREF is an essential heterogeneous nuclear ribonucleoprotein (hnRNP)-like protein that makes a major contribution to the compaction of mRNPs [41]. Yra1 was originally discovered in yeast as a factor with potent RNA-annealing activity [42,43]. Yra1/ALYREF contains a large number of positively-charged residues, and AlphaFold prediction (Figure 2A) indicates that the chain is mainly unfolded except for its RNA recognition motif (RRM) domain. The Yra1/ALYREF RRM domain is considered to be noncanonical because it lacks the RNA sequence-binding specificity that is typically associated with canonical RRMs and showed no affinity for RNA [44]. However, this property may help the Yra1/ALYREF RRM to serve as a prominent protein interaction hotspot in mRNP particles [32]. Yra1/ALYREF contains two copies of a Sub2/UAP56 binding motif, one at each of the N- and C-terminii (UBM-N and UBM-C), that can mediate interactions with Sub2/UAP56 and also Mex67–Mtr2/NXF1–NXT1, such that, in principle, Yra1/ALYREF could bind to both proteins simultaneously [17,45–47]. The evolutionary conservation of this major mRNA-packaging factor points toward a general paradigm governing nuclear mRNP packaging [32]

##### Sub2/UAP56

Sub2/UAP56 is a central component of the TREX complex and functions as a key RNA ATPase involved in facilitating steps such as splicing and transferring mRNPs to the export machinery for exit from the nucleus (reviewed in [48]). Sub2/UAP56 (Figure 2B-D) contains a helicase core formed from two domains that resemble the bacterial recombination protein RecA (RecA1 and RecA2) that are connected via a short linker [48]. The helicase core is flanked by N-terminal (NTE) and C-terminal (CTE) extensions. Like other DEAD-box ATPases, UAP56 utilizes ATP hydrolysis to mediate functions such as remodelling RNA–protein complexes, and has an open conformation in its free, unbound state and a closed conformation when bound on RNA [48]. In the TREX complex, Sub2/UAP56 binds to THO in a semi-open conformation [24,39], but transitions to a closed state upon binding to ATP and mRNA [49]. A conformational change in S. *cerevisiae* Sub2 is thought to facilitate loading of Yra1 onto mRNAs to complete the formation of the TREX complex [39]. In human cells, binding of the TREX complex requires ALYREF that binds to exon–exon junctions by interacting with eIF4A3 in the EJC [33,45]. ALYREF binding is possibly aided by an interaction with the nuclear mRNA cap-binding complex, but is also reported to occur independently of the cap and before exon ligation [22,45,50].

##### THO complex

**Figure 3:**
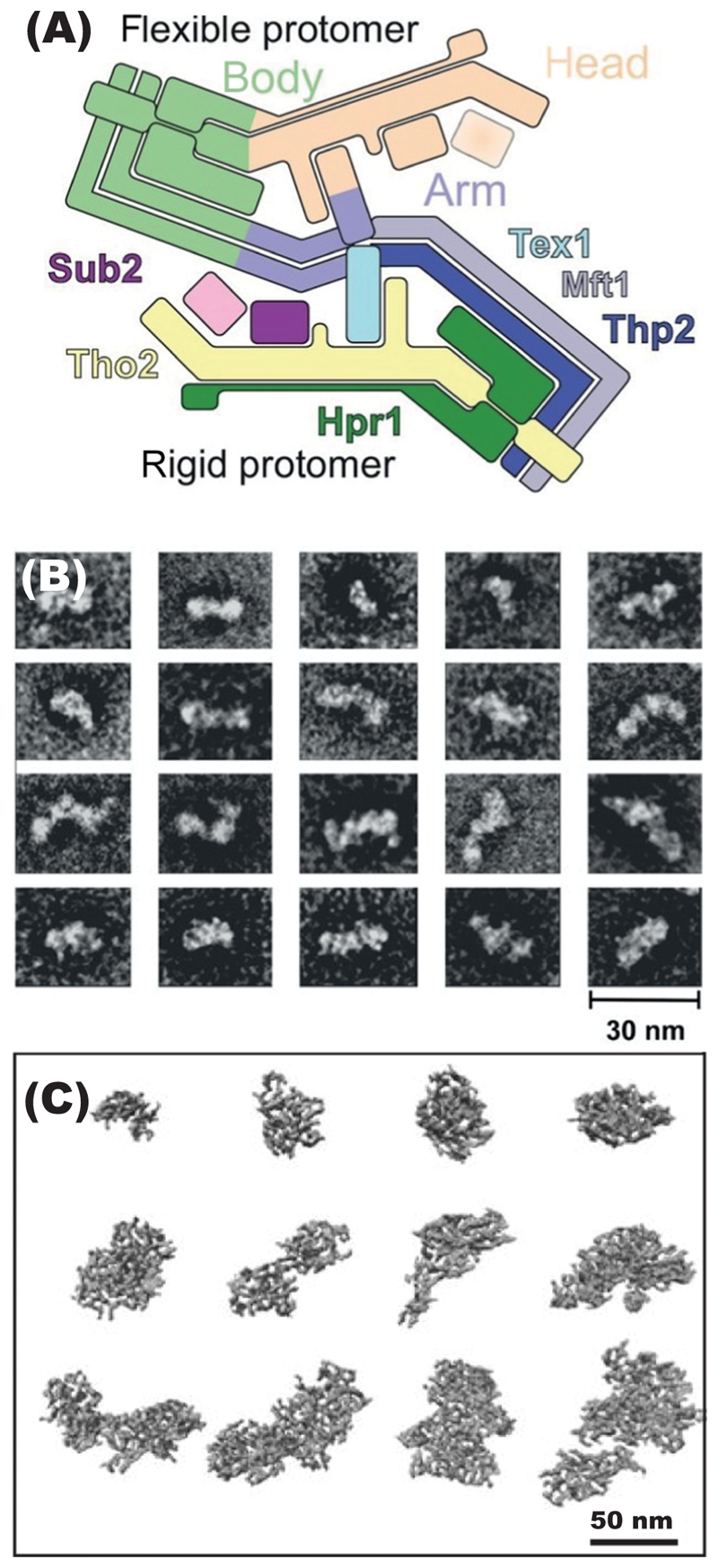
(A) Schematic of the THO complex that forms the body of the *Saccharomyces cerevisiae* TREX complex and to which Yra1 and the DEAD-box ATPase Sub2 bind. Reproduced from [24]. (B) Electron micrographs of negatively stained mature messenger ribonucleoprotein (mRNP) complexes from S. *cerevisiae* showing irregular structures that are often elongated and rod-like with diameters of 12 nm, and where particle length increases with the size of the transcript. Reproduced from [51]. (C) Cryo-electron micrographs of tomograms of selected *S. cerevisiae* TREX complexes showing irregular rod-shaped particles. Reproduced, from [32]. Abbreviation: TREX complex, transcription-export complex.

A 3.4 A resolution cryo-electron microscopy (cryo-EM) structure of a S. *cerevisiae* THO-Sub2 complex reconstituted from expressed proteins [24] showed that it is based on a 20 nm long platform formed by Tho2 and Hpr1 to which Tex1, Mft1, Thp2, and Sub2 attach (Figure 3A). The complex forms dimers in which the THO subunits Tho2 and Hpr1 intertwine to form a platform to which Mft1, Thp2, and Tex1 are bound. The resulting complex homodimerizes in an asymmetric fashion in which a Sub2 molecule is attached to each protomer, and shows a level of flexibility that may contribute to its function [24]. The two Tho2–Hpr1 platforms are arranged in an antiparallel fashion, and the structure can be conveniently described in terms of a body from which two arms or heads extend (Figure 3A). The two Mft1–Thp2 ‘arms’ have coiled-coil structures and extend from opposite ends towards each other to form a chevron-like structure. The homodimerization interfaces appear to be the pivot point around which the remainder of the Mft1–Thp2 coiled-coils and the attached Tho2–Hpr1 platforms swing with a seesaw-like movement that appears to be connected to structural changes in the two Sub2 proteins [24].

Cryo-EM of a THO–Sub2 complex containing the SR protein Gbp2 [25] showed a similar structure, although it did not show the same dimerization. This structure showed how the SR and RRM domains of Gbp2 bind to the THO complex, and this suggested how such an interaction could facilitate loading of Gbp2 onto mRNPs [25].

##### SR proteins

The S. *cerevisiae* SR proteins (Npl3, Gbp2, and Hrb1) are rich in serine and arginine residues and also contain RRM domains (reviewed in [19]). Although they also become attached to the pre-RNA co-transcriptionally, they are not lost when an export-competent mRNP is generated and are instead removed in the cytoplasm following export [19]. The SR proteins also contribute to the compaction of the pre-RNA through binding both pre-RNA and other RBPs and, because of their contribution to mRNA quality control and protection from nucleases, have been described as ‘guard proteins’ [19]. In addition to contributing to compaction, SR proteins also contribute to many of the steps along the nuclear mRNA processing pathway. Npl3, for example, contributes to the addition of the 5′ cap, Gbp2 and Hrb1 contribute to splicing, and Hrb1 also monitors 3′ cleavage [19].

#### Deciphering the structures of mRNPs

##### Isolation of endogenous THO-Sub2/UAP56-containing assemblies

Several studies have examined the arrangement of the TREX components within mature mRNPs in both *S. cerevisiae* [32,40,51 ] and human [33] cells. The yeast studies focused on mature mRNPs that were isolated using affinity methods directed towards Nab2 [51], Hpr1 [40], or Hpr1 plus Sub2 [32].

Bonneau *et al*. [32] purified endogenous mature nuclear mRNPs from *S. cerevisiae* using a bimolecular affinity purification strategy that employed THO and Sub2 as baits and which was optimized to preserve the integrity of these transient assemblies. The THO–Sub2-containing assemblies obtained in this way had the hallmark proteins expected for nuclear mRNPs and contained a broad spectrum of mRNAs, together with THO and Sub2, and known nuclear mRNA-binding proteins such as the 5′ cap-binding protein Cbp80 and the 3′ poly(A) tailbinding protein Nab2. Strikingly, in these preparations there was a considerable over-stoichiometric representation of Yra1 that has also been seen in single-molecule quantification studies [41], indicating that additional Yra1 can bind independently of TREX. These complexes also contained Yhs7 (a currently uncharacterized protein) that has an overall domain organization similar to Yra1, with an RRM domain and positively-charged disordered regions interspersed with small helical segments [41]. mRNAs known to have short half-lives and those containing introns were disproportionately enriched in this material, indicating that these mRNPs were unlikely to derive from the cytoplasmic population. A combination of proteomics, RNA sequencing, cryo-EM, crosslinking mass spectrometry, structural modelling, and biochemical assays indicated that yeast nuclear mRNPs are packaged around an intricate network of interconnected proteins capable of promoting RNA–RNA interactions via their positively-charged intrinsically disordered regions [32]. Cryo-EM images of these nuclear mRNP populations showed a variety of compact, irregular, elongated particles (Figure 3C), whereas negatively stained electron micrographs of this material indicated that the TREX complex is present as a dimer in these particles [32]. A complementary study [40] purified endogenous nuclear mRNPs from S. *cerevisiae* using Hpr1 affinity alone, and again found they contained an excess of Yra1 relative to other TREX complex components.

Pacheco-Fiallos *et al*. [33] showed human nuclear mRNPs that were compacted, although there were several distinct differences from those seen in *S. cerevisiae*. First, the human assemblies exhibited greater uniformity in size and shape compared with the yeast mRNPs, and generally tended to have a roughly spherical shape in which the RNA formed an interior core surrounded by TREX complexes coating the surface of the particle [33]. Second, the human particles presented recognizable THO–UAP56 complexes on their surface. It is currently unclear whether the differences between studies arose, for example, from variations across species or from differences in sample preparation procedures (such as Grafix crosslinking treatment before imaging of the human mRNP samples).

#### Mature mRNP structure

Electron microscopy of purified mRNPs from *S. cerevisiae* [51] showed rod-like structures with a roughly constant width of 5 nm and lengths that increased with molecular weight (Figure 3B); whereas at the other extreme, images of the giant Balbiani ring mRNPs of *Chironomus tentans* showed 50 nm globular structures that became elongated as they traversed through NPCs [52]. Recent single-molecule fluorescence *in situ* hybridization [9,10], proximity ligation studies [11], and *in situ* florescence energy transfer [8] studies on mammalian mRNPs also support mRNPs being dense rod-shaped particles in which the mRNA is compacted. Within these particles, RBPs and EJC proteins likely form a stable scaffolding that nucleates and maintains the mRNA in a densely packaged state. Recent cryo-EM of mRNP particles isolated from *S. cerevisiae* (Figure 3C) also showed they were compacted, had flexible irregular shapes, and were generally elongated [32]. Studies using proximity ligation [11] and/or light microscopy [9,10] were consistent with human mRNPs also having a compacted, flexible, but elongated structure. TREX–EJC–mRNP particles isolated from human cells contained up to three TREX complexes that formed a coat on the mRNP surface [23,33]; they were also more globular, perhaps as a result of containing shorter mRNAs. In these particles, ALYREF multimerized with mRNA-bound EJCs [33] and interacted with UAP56, thereby bridging TREX complexes and EJCs, consistent with previous structural and biochemical data [24,38,39]. However, although UAP56 was present, it was not yet bound to mRNA [33]. Remarkably, although both the *S. cerevisiae* and human particles examined by cryo-EM contained substantial quantities of mRNA, only a few short stretches containing a few nucleotides could be seen attached to the TREX complex in reconstituted human particles, and in *in vitro* particles it was proposed that the bulk of the mRNA adopted multiple conformations that were blurred out in reconstructions that focused on the proteins [33].

### Step 2: the generation of export-competent mRNPs

Export-competent mRNPs are only generated when mature mRNPs have been formed, thereby helping to prevent mRNAs that are incomplete or that have not been processed satisfactorily from being exported to the cytoplasm and translated. In *S. cerevisiae*, the termination of polyadenylation and resultant release of the mRNP from the transcription machinery is a key checkpoint that leads to remodelling of the mRNA and removal of TREX [1,53–55] (discussed further below), as well as the attachment of nuclear export factors Mex67–Mtr2 (Figure 1). However, how the completion of processing is signalled is unclear. The Pcf11 component of the cleavage and polyadenylation machinery appears to be important in this step [56], but precisely how poly(A) polymerase dissociation influences Pcf11 and how this in turn influences TREX remains obscure. In *S. cerevisiae*, there are indications that Mud2, a splicing factor, functions to coordinate splicing and polyadenylation, but the precise mechanism by which this is mediated and the role of other factors that show genetic interactions between these functions is unclear [2,14]. mRNAs that still retain introns appear to be retained in the nucleus, either at the nuclear basket (Box 1) in *S. cerevisiae* or in nuclear speckles in metazoans; although several components of the machinery involved have been identified [57], the precise mechanism by which this is mediated remains unclear.

Termination of polyadenylation appears to be mediated by dissociation of poly(A) polymerase from the cleavage and polyadenylation machinery as a result of steric factors associated with the binding of Nab2 in budding yeast and PABPN1 in higher eukaryotes. In budding yeast, Nab2 controls poly(A) tail length [14] and probably mediates this function though dimer formation [58], but the *in vivo* structure of Nab2 dimers bound to poly(A) mRNA and whether there are one or more dimers per tail remain to be established. *In vitro*, PABPN1 forms globular aggregates on poly(A) mRNA [59] that are proposed to eventually force the dissociation of poly(A) polymerase (reviewed in [60]), but the precise structure of these aggregates remains to be established to define how many PABPN chains each contains and how the chains are arranged to generate a defined particle size. Although polyadenylation is terminated by the dissociation of poly(A) polymerase from the cleavage and polyadenylation machinery, how this machinery detaches from the 3′ untranslated region (UTR) is less clear. It may be that the attachment of poly(A) polymerase to the 3′ UTR is sufficiently weak to simply dissociate, but it seems more likely that dissociation of poly(A) polymerase generates some conformation change and/or post-transcriptional modification [such as phosphorylation or the attachment of small ubiquitin-like proteins (SUMOylation)] of the complex that could also facilitate its release, although detailed evidence for this point is lacking.

It is not completely clear how the completion of mRNP maturation is recognized to initiate the generation of an export-competent mRNP. It has been proposed that the ubiquitin ligase Tom1 [2,31,61] and Arg methylation [62,63] may contribute to initiating the remodelling though modification of Yra1/ALYREF. Another possibility is that release from the transcription, splicing, and polyadenylation machinery is, in itself, a signal that a mature mRNP has been generated. In yeast, it may be that this release enables binding of the mRNP to the nuclear basket where the concentration of Mex67–Mtr2 complex is high because of its affinity for the Phe/Gly-motif (FG) nucleoporins that fill the NPC transport channel; indeed, it appears that only NPC-associated Mex67, and not soluble Mex67 in the nucleoplasm, is necessary to mediate nuclear export [64]. Light microscopy shows that mature mRNAs meander along the inner nuclear envelope for some time before becoming bound to a NPC, after which they are rapidly exported [65]. In S. *cerevisiae* the initial binding of the mRNP to NPCs appears to be facilitated by Nab2 bound to the poly(A) tail, and which is able to interact with the nuclear basket component Mlp1 and Mex67–Mtr2 [66]. Mature mRNPs may also bind to the TREX2 complex that can bind to mRNA and is also located at the nuclear basket [67] (discussed further below).

#### Removal of the TREX complex

**Figure 4:**
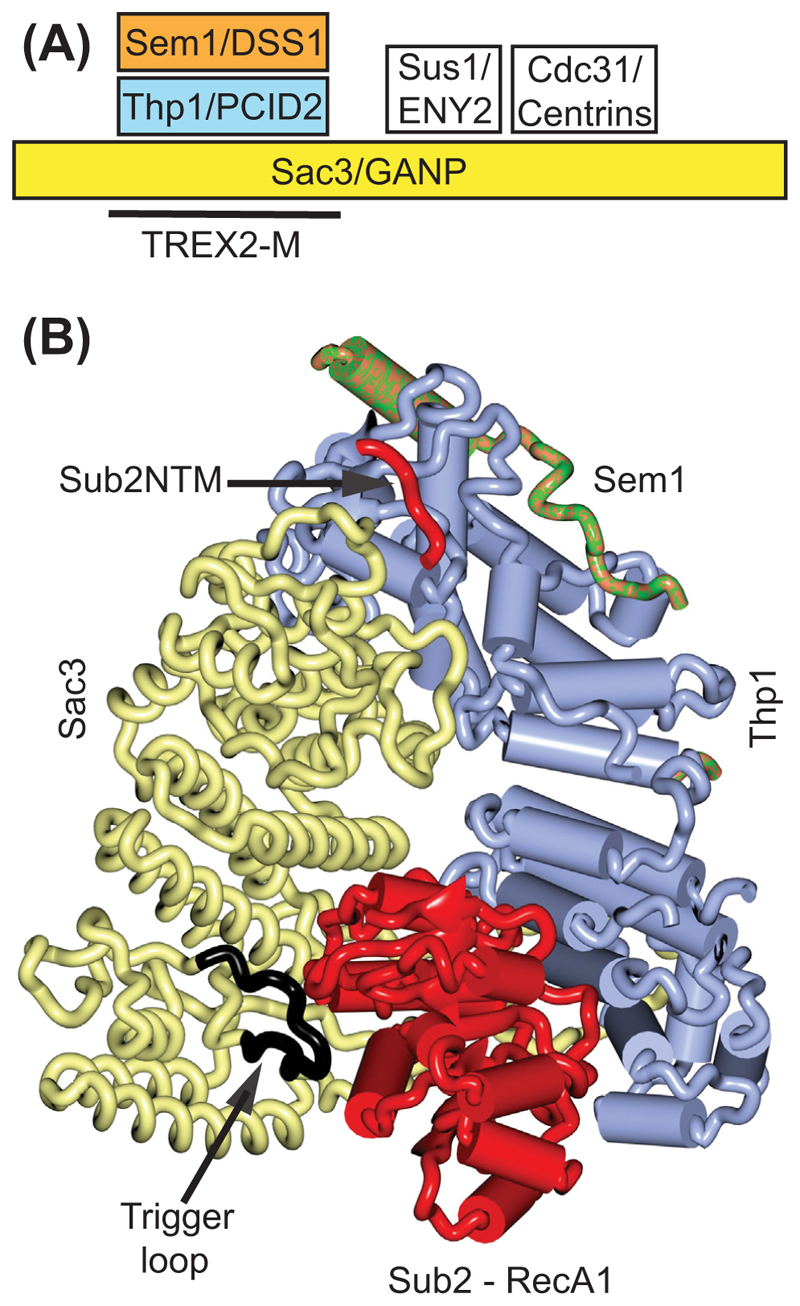
TREX2 complex and its interaction with Sub2. (A) Schematic of the TREX2 complex that is based on a core of Sac3/GANP to which Thp1/PCID2, Sus1/ENY2, and Cdc31/contrins bind. The central M region, TREX2-M, has a ‘V’ shape (B) to which Sub2 (red) binds, with its NTM at the tip of the ‘V’ formed by Sac3 and Thp1, and the RecA1 domain sandwiched between them lower down. In addition, a ‘trigger’ loop (black), that is disordered in the structure of TREX2 alone, becomes ordered and attached to the RecA1 domain in a position corresponding to the ATP-binding site and which would clash with the RecA2 domain in the closed conformation. Based on PDB 8U8E [68]. Abbreviations: NTM, N-terminal motif; TREX complex, transcription-export complex.

Recent work on several *in vitro* complexes [68–70] indicated that Tho1/SARNP and the TREX2 complex appear to make a major contribution to the remodelling that removes the TREX complex and attaches Mex67–Mtr2/NXF1–NXT1 to generate an export-competent mRNP. Tho1/SARNP has KxxxRxxR/KFG sequence motifs (two and five, respectively) that bind to the N- and C- terminal regions of Sub2/UAP56. Because these Sub2/UAP56 regions also bind to the THO complex, attachment of Tho1/SARNP to the mature mRNP has the potential to destabilize the attachment of THO to other components of the TREX complex and thus contribute to its removal [68,71]. Although it was known that the TREX2 complex participates in mRNA export [72], the stage at this was mediated was unclear (reviewed in [73]). Recent work [68,69] indicates that the TREX2 complex, located primarily at the nuclear basket, and also a related TREX2-1 complex located in nuclear speckles, stimulates the Sub2/UAP56 ATPase, resulting in its removal from the mRNP; extensive work supporting this mechanism has also been reported in a preprint [70]. The TREX2 complex (Figure 4A) is based on a scaffold of Sac3/GANP to which Thp1/PCID2, Sus1/ENY2, and Cdc31/centrins are bound [67,73–75], whereas TREX2-1 is based on an analogous LENG8 scaffold [69]. The middle module of TREX2 and TREX2-1 (TREX2M and TREX2-1M) that contains Thp1/PCID2 and Sem1/DSS1 bound to the central region of Sac3/GAMP/LENG8 (Figure 4B) is able to bind to Sub2/UAP56 [68,69]. In both human or *S. cerevisiae* systems, addition of TREX2M to a preassembled Sub2/UAP56–U_10_RNA complex, with or without bound Tho1/SARNP, triggered displacement of Sub2/UAP56 from the RNA. Although UAP56 unloading did not depend on ATP hydrolysis, TREX2M activated the ATPase activity of Sub2/UAP56 substantially [68,69]. Pull-down assays with purified proteins identified the N-terminal motif (NTM) of Sub2/UAP56 as the major binding site for TREX2M [68].

A crystal structure of the S. *cerevisiae* TREX2M–Sub2NTM complex showed residues 10–20 of the Sub2NTM at the tip of the 'V' formed by Sac3 and Thp1 [68]. At the TREX2M interface with Sub2NTM, both Sac3 and Thp1 are enriched in positively-charged residues, whereas Sub2NTM is highly enriched in conserved negatively charged residues. The primary role of Sub2NTM appears to tether Sub2 to TREX2, allowing TREX2 to influence its RecA domains and promote Sub2 release from the mRNP. In addition, a cryo-EM structure of a crosslinked S. *cerevisiae* TREX2M–Sub2 complex [68] showed that the Sub2 RecA1 domain is sandwiched between Sac3 and Thp1 (Figure 4B) and, although the RecA2 domain was disordered in this structure, in the human complex it was located next to the RecA1 domain on Sac3/GANP [68,69]. There was an extensive interface between Sub2RecA1 and TREX2M and, strikingly, an extended ‘trigger’ loop in Sac3 (residues 239–252) – that was disordered in structures of TREX2M – was now positioned to occupy the nucleotide-binding pocket between the two Sub2 RecA domains of Sub2 in a way that would introduce steric clashes between the RecA1 and RecA2 lobes in RNA-bound Sub2. This steric clash could destabilize the interactions between them and trigger Sub2 release from RNA, thereby promoting the nucleotide-related rate-limiting steps of Sub2 [68,73], a hypothesis that was supported by mutagenesis of the trigger loop. A similar influence of the TREX2M region was described in analogous UAP56 complexes [69,70] where a similar ordering of a GANP/LENG8 ‘trigger’ loop between the UAP56 RecA1 and RecA2 domains was observed, consistent with TREX2 making a major contribution to orchestrating the removal of TREX components from the mRNP [69]. It appears that mRNPs influenced by TREX2-1 have a higher CG content and a greater number of introns than those influenced by TREX2 [69,75]. However, although both the TREX2 and TREX2-1 complexes mediate the displacement of Sub2/UAP56 from mRNPs, the detailed series of steps that generate export-competent mRNPs have not yet been defined.

#### Attaching the Mex67–Mtr2/NXF1–NXT1 transport factor

Although several RBPs, including Yra1/ALYREF and some SR proteins such as Npl3, appear into interact with Mex67–Mtr2/NXF1–NXT1 and attach it loosely to mRNPs, it appears to only become firmly attached following the Sub2/UAP56-orchestrated remodelling of the mRNA that possibly involves a change in mRNA secondary structure [2,76,77], together with Yra1 ubiquitinylation by theTom1 E3 ligase [61]. This attachment appears to occur mainly at the NPC nuclear face [68]. In addition to binding to RNA, the Mex67–Mtr/NXF1–NXT1 transport factor complex contains domains that bind to the FG regions of nucleoporins that pack the NPC central transport channel and so can overcome the barrier produced by this crowded environment, thereby facilitating movement of the mRNA backwards and forwards through the channel [1,53,55,78–81].

**Figure 5:**
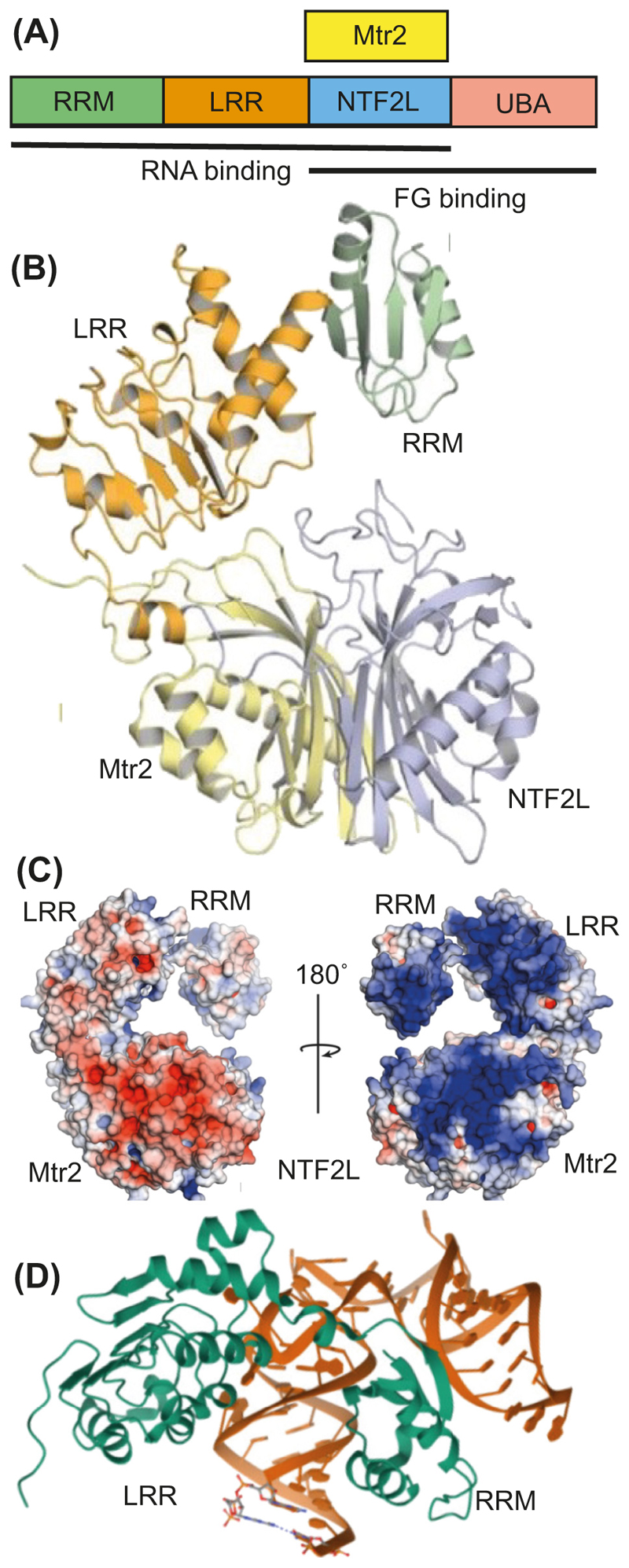
Schematic (A) and structure (B) of the Mex67-Mtr2 nuclear export factor. Mex67 has RRM, LRR, NTF2-like, and UBA domains that can bind to mRNAs and FG-nucleoporins. (C) One surface of Mex67-Mtr2 has a considerable overall positive charge (blue) that can complement the negative charge of the RNA phosphate backbone, whereas the opposite side has sites that bind to Phe/Gly (FG) repeats. Reproduced from [81]. (D) Crystal structure showing the binding of the LRR and RRM domains of the human Mex67 homolog NXF1 to stem-loops in simian type D retrovirus CTE mRNA (from PDB 3RW6 [80]). Abbreviations: CTE, constitutive transport element; LRR, leucine-rich repeat; RRM, RNA recognition motif; UBA, ubiquitin-associated.

Mex67 contains four domains (Figure 5A,B). The RRM and leucine-rich repeat (LRR) domains can bind to RNA, whereas the nuclear transport factor 2 (NTF2)-like domain binds to Mtr2 (that also has a NTF2-like structure) and both the ubiquitin-associated (UBA) and NTF2-like domains are able to bind to FG nucleoporin repeats [1,53,55,78–81]. Mex67–Mtr2 may dimerize to generate a large positively-charged platform (Figure 5C) to facilitate its binding to mRNA [81], primarily through interactions of its large positively-charged face with the phosphate backbone. Unlike karyophenin-based protein import and export factors (reviewed in [79,82]), Mex67–Mtr2/NXF1-NXT1 does not appear to bind to a specific nucleotide sequence analogous to a NLS (nuclear localization sequence) or NES (nuclear export sequence). However, the structure of a complex of the RRM and LRR domains of NXF1 complexed with simian type D retrovirus constitutive transport element (CTE) mRNA (Figure 5D) shows extensive interactions with stem-loops [80], suggesting that the conformation (secondary/tertiary structure) of a mRNA could contribute to its recognition. In the NXF1 RRM–LRR complex, basic and hydroxyl-containing side chains of the RRM domain form sugar-phosphate backbone contacts with the CTE RNA, whereas base-edge and sugar-phosphate backbone contacts are formed with loop elements that project from a face of the LRR domain. Although CTE RNA appears to bind NXF1–NXT1 more strongly than cellular mRNAs, it is likely that they employ a similar mechanism, but details are currently lacking. An analogous Mex67–Mtr2 interaction with pre-60S ribosomal particles has been observed [83] where the RNA conformation, together with other proteins, appears to be closely associated with the interaction.

It is not known precisely how Sub2/UAP56 is able to facilitate the binding of Mex67–Mtr2/NXF1–NXT1, although the ability of DEAD-box ATPases to modify RNA secondary structure would make a mechanism based on this property an attractive possibility and would be consistent with the central role of DEAD-box ATPases in both loading Mex67–Mtr2/NXF1–NXT1 in the nucleus (to facilitate passage through NPCs) and its removal at the cytoplasmic face (to prevent return to the nucleus) [84,85]. It is possible that the mRNA structure generated to facilitate binding to Mex67–Mtr2/NXF1–NXT1 is metastable and, because forming it consumes energy, given the opportunity, it would revert to the unbound conformation. In this scenario, once released at the cytoplasmic face, Mex67–Mtr2/NXF1–NXT1 would not rebind.

The removal of TREX when generating an export-competent complex may make mRNPs more flexible to facilitate movement through the NPC [8], and it is also possible that protection from nuclear nucleases is no longer required. Remodelling also removes the need to recycle many of the accessory proteins.

### Steps 3 and 4: mRNA nuclear export through the NPC and release into the cytoplasm

Following proper processing of the mRNA and the acquisition/removal of specific RBPs, export-competent mRNPs travel from the nucleus to the cytoplasm through NPCs. The Mex67–Mtr2/NFX1–NXT1 [18] complex mediates movement of mRNPs back and forth in the NPC through its interactions with the FG repeat cores of the natively unfolded regions of FG-nucleoporins that pack the central transport channel (Box 1), thereby overcoming the barrier they generate [86,87]. At the cytoplasmic face of the NPC, mRNPs encounter Gle1, Nup42, and Nup214, that activate the DEAD-box ATPase Dbp5/DDX19B that mediates the removal of Mex67–Mtr2/NXF1–NXT1 from the mRNP [54,88–95]. Removal of Mex67–Mtr2/NXF1–NXT1 prevents the mRNA from returning to the nucleus, and thus provides directionality to mRNA export together with freeing Mex67–Mtr2/NXF1–NXT1 to return to the nucleus to mediate additional rounds of mRNP export. Energetically, this process is an example of a thermal ratchet [96] whereby the energy liberated by ATP cleavage by Dbp5/DDX19B is able to rectify the thermal motion of the mRNA [97].

#### Dpb5/DDX19B remodelling of the mRNP at the NPC cytoplasmic face

The binding of Gle1/GLE1 and Nup159/NUP214 generates a conformational change in Dbp5/DDX19B that opens the nucleotide-binding pocket, facilitating exchange of ADP for ATP and increasing its affinity for mRNA [91,98–100]. In S. *cerevisiae*, inositol hexakisphosphate (IP6) also contributes to this process, although its role regarding human DDX19B is less clear [100]. Like Sub2 (Figure 2C,D), Dbp5/DDX19B contain two RecA domains that are joined by a flexible linker [98,101,102], and binding of mRNA and ATP transitions these domains from open to closed conformations, generating an extended RNA-binding interface that is thought to induce a kink in the RNA structure [103]. When the Gle1–Dbp5/DDX19B complex binds to mRNA at the NPC cytoplasmic face, it is thought that the formation of the closed catalytically active state of Dbp5/DDX19B triggers the release of Gle1 and ATP hydrolysis that ultimately lead to local remodelling of the mRNA, resulting in displacement of Mex67–Mtr2/NXF1–NXT1 [84,85,90,95,100,104], although the mechanism by which this is achieved remains unclear. It is not known how Dbp5 targets the specific region of RNA to modify the binding of Mex67–Mtr2/NXF1–NXT1, or Nab2 in *S. cerevisiae*, whereas some other mRNP components remain associated to function in the cytoplasm, but it is likely that protein–protein interactions are important. In *S. cerevisiae*, Dbp5 associates in an RNA-dependent manner specifically with Nab2 and Mex67 at the NPC, and Dbp5 localization at the NPC is sufficient to direct mRNP remodelling and support cell viability [90]. Dbp5 interaction with RNA is not required for its association with Mex67, and becoming associated with the mRNP before export is not required for its remodelling activity at the NPC [90]. In *S. cerevisiae*, the recruitment of Dbp5 to remodel the mRNA structure in the proximity of Mex67 and Nab2 suggests that these proteins might associate in a complex, but interactions have not been detected *in vitro* between recombinant Dbp5, Mex67–Mtr2, Nab2, and Gle1 in the presence of RNA, which could indicate that additional factors (such as additional proteins or post-translational modifications) are required for Dbp5 association with Nab2 and/or Mex67, or that the Nab2–Dbp5 and/or Mex67–Dbp5 association *in vivo* is transient and leads directly to mRNP remodelling [90].

Overall, remodelling of the export-competent mRNP by Dbp5/DDX19B releases the mRNA into the cytoplasm for translation as well as preventing its return to the nucleus. This completes the nuclear journey of the mRNA from transcription, capping, splicing, polyadenylation and cleavage to release into the cytoplasm for translation.

## Concluding remarks

Although the mechanisms by which transport factors mediate the transport of mature mRNAs across the nuclear envelope through NPCs and the machinery by which transport factors are released in the cytoplasm are understood in some detail, the structure and even the stoichiometry of any particular mRNP remains unclear. Major unresolved issues include how mRNAs are remodelled to bind and release transport factors and RBPs, and how successful completion of the nuclear mRNA processing segment of the gene expression pathway is signalled to initiate the generation of export-competent mRNPs (see Outstanding questions).

To enable the export of mRNA through nuclear pores to the cytoplasm, it is necessary both to add nuclear export factors and to release the mRNA from RNA polymerase and the nuclear processing machinery. After cleavage in the 3′ UTR, the RNA polymerase is still probably attached to the mRNA though interactions between its C-terminal domain (CTD) and the cleavage and polyadenylation machinery that are likely retained until polyadenylation has been completed. Similarly, retention factors appear to impair export until splicing has been completed. Both splicing and polyadenylation appear to be coordinated with the attachment of nuclear export factors, but the temporal sequence of events remains unclear. Moreover, these steps may not necessarily need to occur in a defined sequence, and instead export may only occur when all have been completed. Although most of the factors involved in the generation of export-competent mRNPs have been identified, the precise mechanism by which they function to coordinate the different steps of the nuclear processing machinery and the way in which the various factors interact with mRNA are less clear. Key aspects that remain to be clarified include the role of mRNA conformation together with the details of how Yra1/ALYREF, TREX, and Mex67–Mtr2/NXF1–NXT1 bind to mRNA, the way in which DEAD-box ATPases remodel mRNPs, and also the signal that pre-mRNA processing has been completed to initiate remodelling of the mature mRNP to make it export-competent. Although attachment of nuclear export factors is a prerequisite for movement of mRNAs through nuclear pores to the cytoplasm, it remains unclear how many of these factors are attached to an individual mRNA.

In summary, many of the components of the gene expression pathway that are involved in the nuclear processing and export of mRNAs have been identified, but the precise mechanisms by which many of these steps are coordinated remain to be established. Although the thermal ratchet-based mechanism by which mRNPs are exported to the cytoplasm though NPCs has been established in considerable detail, many aspects of the generation of export-competent mRNPs in the nucleus remain unclear. Key checkpoints in the pathway to the generation of export-competent mRNPs, that entail establishing that both splicing and polyadenylation have been completed, need to be activated in a timely manner. Providing a comprehensive description of this complex series of integrated processes represents an important challenge and a fruitful area for future studies in this area.

## Highlights

The passage of mRNAs through nuclear processing and export to the cytoplasm is mediated by a spectrum of RNA-binding proteins and detailed information about their structure and the way in which they interact with mRNAs to generate messenger ribonucleoprotein complexes (mRNPs) is emerging.

Mature mRNPs, in which processing has been completed, are compacted by the transcription-export (TREX) complex and Ser/Arg-rich proteins, after which they are remodeled to facilitate export through nuclear pores to the cytoplasm for translation.

DEAD-box ATPases mediate much of the extensive remodelling of mRNPs in this pathway that is necessary both to generate export-competent mRNPs that contain nuclear export factors and to remove these factors in the cytoplasm to prevent the mRNA returning to the nucleus.

The TREX2 complex and Tho1/SARNP orchestrate key features of the remodelling required to produce an export-competent mRNP.

## Glossary

### Abbreviations

Key components of the mRNA export machinery in the Glossary are formatted as Yeast/METAZOAN.

Dbp5/DDX19B: DEAD-box ATPase–mRNP export complex disassembly protein.
Exon-junction complex (EJC): a macromolecular complex that binds near to exon junctions and recruits the THO complex in metazoans.
Gle1/GLE1: enhances Dbp5/DDX19 activity
Mex67–Mtr2/NXF1–NXT1: an mRNA export factor complex that facilitates transport through nuclear pore complexes (NPCs). Also called TAP–P15 in metazoans.
Nup159/NUP214: a nucleoporin that is present on the NPC cytoplasmic face.
Sac3/GANP: the scaffold of the TREX2 complex.
Spliceosome: a large macromolecular complex that mediates splicing.
Ser/Arg-rich (SR) proteins: proteins that aid compaction.
Sub2/UAP56 (DDX39B): aTREX component and DEAD-box ATPase.
THO complex: a TREX component.
Tho1/SARNP: an RNA-binding protein (RBP) that binds to Sub2/UAP56 in the TREX complex and facilitates its dissociation from mature mRNPs.
Transcription-export (TREX) complex: a complex that aids mRNP formation.
TREX2 complex: guides mRNPs to NPC nuclear basket and aids the generation of export-competent mRNPs.
Yra1/ALYREF: a TREX component.

## Box 1 Nuclear transport of macromolecules

To maintain the distinctive composition of each compartment, the selective movement of macromolecules larger than 40 kDa is mediated by nuclear pore complexes (NPCs). These huge macromolecular assemblies have a circular body, from which filaments project into the cytoplasm, and afibrous nuclear basket that projects into the nucleus (Figure IA) and have a central transport channel densely packed with disordered regions of nuclear pore proteins (FG-nucleoporins) that contain multiple phenylalanine/glycine (FG) repeat motifs (Figure IB). This crowded environment forms a barrier that impedes the movement of macromolecules larger than 40 kDa (reviewed in [82,105,106]). The movement of these macromolecules through NPCs is facilitated by a range of different carrier proteins, although the pathways are all examples of active transport and are based on a thermal ratchet mechanism [96] in which energy is used to rectify Brownian motion by mediating the assembly of cargo:carrier complexes in the donor compartment and their disassembly in the acceptor compartment [2]. For example, karyopherin-based protein import and export is powered by Ran GTP hydrolysis [82], whereas mRNA export (except for some small mRNAs and some viral RNAs that use Xpo1/CRM1 together with adapter proteins such as HIV REV) uses Mex67–Mtr2/NXF1–NXT1 and energy derived from ATP hydrolysis by DEAD-box ATPases [53,79].

## Box 2 Conformation of mRNA

Although mRNAs are generated as linear polymers by the transcription machinery, they generally have an inherent tendency to fold into relatively compact particles and can easily form local double-stranded structures in which the chain folds over on itself to form hairpin stem-loops and related structures that are stabilized by intramolecular hydrogen bonds formed through complementary base-pairing [107]. These secondary structures often then fold into 3D tertiary structures. However, unlike tRNAs and many noncoding RNAs that adopt defined structures [108], mRNAs may not adopt a single defined structure and transcripts instead transition between several different related structures that have roughly the same overall energy with the activation energy for moving between these structures being relatively small [108]. Because the mRNA will fold as it is synthesized in a 5′ to 3′ direction, the conformations adopted by molecules generated in this way could potentially differ from those generated when the whole transcript refolds in solution, and it is possible that the pre-mRNAs generated by transcription may not represent the lowest energy state of the chain, especially if particular conformations are stabilized by the binding of nuclear RNA-binding proteins (RBPs). Moreover, proteins binding to the transcript probably stabilize particular mRNA secondary/tertiary structures and increase the barrier to transitioning to another, possibly lower-energy, structure. Although some of the proteins involved in splicing and cleavage/polyadenylation recognize specific RNA sequences, other proteins (the TREX complex components Yra1/ALYREF and Sub2/UAP56 or the Mex67–Mtr2/NXF1–NXT1 export complex, for example [2,53]) appear not to recognize sequences and may instead bind to and/or mediate the formation of specific secondary and tertiary mRNA structures [109]. RNA secondary and tertiary structures are frequently stabilized by RBPs – as can be seen, for example, in the structure of ribosomes and other RNPs. One way in which proteins can facilitate the generation of mRNP structural features is by facilitating the formation of stem-loops by providing sequences rich in positively-charged residues that can contribute to neutralizing the high concentration of negatively charged phosphates that are generated in antiparallel double-stranded stems. Yra1 (**y**east **R**NA **a**nnealing), for example, was initially identified through the way in which it facilitates the formation of an antiparallel dimer from two complementary RNAs [42].

## Outstanding questions

How do DEAD-box ATPases such as Sub2/UAP56 and Dbp5/DDX19B remodel mRNPs to facilitate the binding and release of proteins?

How do different proteins in the gene expression pathway interact with mRNAs?

What is the role of mRNA conformation in the binding and release of RBPs and transport factors?

What is the signal that nuclear processing has been completed and that the generation of an export-competent mRNP should commence?

Are the structure and composition of the mRNPs generated for a particular transcript always the same?

What are the detailed pathway by which TREX2 and TREX2-1 complexes generate export-competent mRNPs?

How are different steps of the gene expression pathway coordinated?

## References

[1] Chen S (2024). Nuclear mRNA export. Acta Biochim Biophys Sin Shanghai.

[2] Stewart M (2019). Polyadenylation and nuclear export of mRNAs. J Biol Chem.

[3] Andersen CB (2006). Structure of the exon junction core complex with a trapped DEAD-box ATPase bound to RNA. Science.

[4] Bono F (2006). The crystal structure of the exon junction complex reveals how it maintains a stable grip on mRNA. Cell.

[5] Wilkinson ME (2020). RNA splicing by the spliceosome. Annu Rev Biochem.

[6] Perez-Calero C (2020). UAP56/DDX39B is a major cotranscriptional RNA-DNA helicase that unwinds harmful R loops genome-wide. Genes Dev.

[7] Shen J (2007). Biochemical characterization of the ATPase and helicase activity of UAP56, an essential pre-mRNA splicing and mRNA export factor. J Biol Chem.

[8] Ashkenazy-Titelman A (2022). RNA export through the nuclear pore complex is directional. Nat Commun.

[9] Adivarahan S (2018). Spatial organization of single mRNPs at different stages of the gene expression pathway. Mol Cell.

[10] Khong A, Parker R (2020). The landscape of eukaryotic mRNPs. RNA.

[11] Metkar M (2018). Higher-order organization principles of pre-translational mRNPs. Mol Cell.

[12] Kataoka N (2023). The nuclear cap-binding complex, a multitasking binding partner of RNA polymerase II transcripts. J Biochem.

[13] Boreikaite V, Passmore LA (2023). 3-End processing of eukaryotic mRNA: machinery, regulation, and impact on gene expression. Annu Rev Biochem.

[14] Soucek S (2012). The long and the short of it: the role of the zinc finger polyadenosine RNA binding protein, Nab2, in control of poly(A) tail length. Biochim Biophys Acta.

[15] Kuhn U (2017). The nuclear poly(A) binding protein of mammals, but not of fission yeast, participates in mRNA polyadenylation. RNA.

[16] Kuhn U (2009). Poly(A) tail length is controlled by the nuclear poly(A)-binding protein regulating the interaction between poly(A) polymerase and the cleavage and polyadenylation specificity factor. J Biol Chem.

[17] Strasser K (2002). TREX is a conserved complex coupling transcription with messenger RNA export. Nature.

[18] Katahira M (1999). Structural study of an RNA aptamer for a Tat protein complexed with ligands. Nucleic Acids Symp Ser.

[19] Querl L, Krebber H (2024). Defenders of the transcriptome: guard protein-mediated mRNA quality control in *Saccharomyces cerevisiae*. Int J Mol Sci.

[20] Zuckerman B (2020). Gene architecture and sequence composition underpin selective dependency of nuclear export of long RNAs on NXF1 and the TREX complex. Mol Cell.

[21] Viphakone N (2012). TREX exposes the RNA-binding domain of Nxf1 to enable mRNA export. Nat Commun.

[22] Viphakone N (2019). Co-transcriptional loading of RNA export factors shapes the human transcriptome. Mol Cell.

[23] Puhringer T (2020). Structure of the human core transcription-export complex reveals a hub for multivalent interactions. Elife.

[24] Schuller SK (2020). Structural insights into the nucleic acid remodeling mechanisms of the yeast THO-Sub2 complex. Elife.

[25] Xie Y (2021). Cryo-EM structure of the yeast TREX complex and coordination with the SR-like protein Gbp2. Elife.

[26] Jensen TH (2001). The DECD box putative ATPase Sub2p is an early mRNA export factor. Curr Biol.

[27] Strasser K, Hurt E (2001). Splicing factor Sub2p is required for nuclear mRNA export through its interaction with Yra1p. Nature.

[28] Stutz F (2000). REF, an evolutionary conserved family of hnRNP-like proteins, interacts with TAP/Mex67p and participates in mRNA nuclear export. RNA.

[29] Lei EP (2001). Messenger RNAs are recruited for nuclear export during transcription. Genes Dev.

[30] Zenklusen D (2002). Stable mRNP formation and export require cotranscriptional recruitment of the mRNA export factors Yra1p and Sub2p by Hpr1p. Mol Cell Biol.

[31] Abruzzi KC (2004). Biochemical analysis of TREX complex recruitment to intronless and intron-containing yeast genes. EMBO J.

[32] Bonneau F (2023). Nuclear mRNPs are compact particles packaged with a network of proteins promoting RNA-RNA interactions. Genes Dev.

[33] Pacheco-Fiallos B (2023). mRNA recognition and packaging by the human transcription-export complex. Nature.

[34] Cheng H (2006). Human mRNA export machinery recruited to the 5’ end of mRNA. Cell.

[35] Clarke BP (2024). Cryo-EM structure of the CBC–ALYREF complex. Elife.

[36] Taniguchi I, Ohno M (2008). ATP-dependent recruitment of export factor Aly/REF onto intronless mRNAs by RNA helicase UAP56. Mol Cell Biol.

[37] Shi M (2017). ALYREF mainly binds to the 5’ and the 3’ regions of the mRNA in vivo. Nucleic Acids Res.

[38] Dufu K (2010). ATP is required for interactions between UAP56 and two conserved mRNA export proteins, Aly and CIP29, to assemble the TREX complex. Genes Dev.

[39] Ren Y (2017). Structural and biochemical analyses of the DEAD-box ATPase Sub2 in association with THO or Yra1. Elife.

[40] Kern C (2023). Cross-linking mass spectrometric analysis of the endogenous TREX complex from *Saccharomyces cerevisiae*. RNA.

[41] Asada R (2023). Single-molecule quantitation of RNA-binding protein occupancy and stoichiometry defines a role for Yra1 (Aly/REF) in nuclear mRNP organization. Cell Rep.

[42] Portman DS (1997). YRA1, an essential *Saccharomyces cerevisiae* gene, encodes a novel nuclear protein with RNA annealing activity. RNA.

[43] Infantino V, Stutz F (2020). The functional complexity of the RNA-binding protein Yra1: mRNA biogenesis, genome stability and DSB repair. Curr Genet.

[44] Zenklusen D (2001). The yeast hnRNP-Like proteins Yra1p and Yra2p participate in mRNA export through interaction with Mex67p. Mol Cell Biol.

[45] Gromadzka AM (2016). A short conserved motif in ALYREF directs cap- and EJC-dependent assembly of export complexes on spliced mRNAs. Nucleic Acids Res.

[46] Heath CG (2016). The role of TREX in gene expression and disease. Biochem J.

[47] Le Hir H (2000). The spliceosome deposits multiple proteins 20-24 nucleotides upstream of mRNA exon-exon junctions. EMBO J.

[48] Yellamaty R, Sharma S (2024). Critical cellular functions and mechanisms of action of the RNA helicase UAP56. J Mol Biol.

[49] Chen C (2021). Structural and functional insights into R-loop prevention and mRNA export by budding yeast THO-Sub2 complex. Sci Bull (Beijing).

[50] Cordiner RA (2023). Temporal-iCLIP captures co-transcriptional RNA-protein interactions. Nat Commun.

[51] Batisse J (2009). Purification of nuclear poly(A)-binding protein Nab2 reveals association with the yeast transcriptome and a messenger ribonucleoprotein core structure. J Biol Chem.

[52] Skoglund U (1983). Visualization of the formation and transport of a specific hnRNP particle. Cell.

[53] De Magistris P (2021). The great escape: mRNA export through the nuclear pore complex. Int J Mol Sci.

[54] Lund MK, Guthrie C (2005). The DEAD-box protein Dbp5p is required to dissociate Mex67p from exported mRNPs at the nuclear rim. Mol Cell.

[55] Valkov E (2012). Structural basis for the assembly and disassembly of mRNA nuclear export complexes. Biochim Biophys Acta.

[56] Johnson SA (2009). Cotranscriptional recruitment of the mRNA export factor Yra1 by direct interaction with the 3’ end processing factor Pcf11. Mol Cell.

[57] Hackmann A (2014). Quality control of spliced mRNAs requires the shuttling SR proteins Gbp2 and Hrb1. Nat Commun.

[58] Aibara S (2017). Structural basis for the dimerization of Nab2 generated by RNA binding provides insight into its contribution to both poly(A) tail length determination and transcript compaction in *Saccharomyces cerevisiae*. Nucleic Acids Res.

[59] Keller RW (2000). The nuclear poly(A) binding protein, PABP2, forms an oligomeric particle covering the length of the poly(A) tail. J Mol Biol.

[60] Wahle E, Ruegsegger U (1999). 3’-End processing of pre-mRNA in eukaryotes. FEMS Microbiol Rev.

[61] Iglesias N (2010). Ubiquitin-mediated mRNP dynamics and surveillance prior to budding yeast mRNA export. Genes Dev.

[62] Lesbirel S (2018). The m^6^A–methylase complex recruits TREX and regulates mRNA export. Sci Rep.

[63] Hung ML (2010). Arginine methylation of REF/ALY promotes efficient handover of mRNA to TAP/NXF1. Nucleic Acids Res.

[64] Derrer CP (2019). The RNA export factor Mex67 functions as a mobile nucleoporin. J Cell Biol.

[65] Saroufim MA (2015). The nuclear basket mediates perinuclear mRNA scanning in budding yeast. J Cell Biol.

[66] Fasken MB (2008). Functional significance of the interaction between the mRNA-binding protein, Nab2, and the nuclear pore-associated protein, Mlp1, in mRNA export. J Biol Chem.

[67] Jani D (2014). Structural basis for binding the TREX2 complex to nuclear pores, GAL1 localisation and mRNA export. Nucleic Acids Res.

[68] Xie Y (2025). Structures and mRNP remodeling mechanism of the TREX-2 complex. Structure.

[69] Clarke BP (2025). Structure-based mechanism of DDX39B regulation by human TREX-2 and a related complex in mRNP remodeling and nuclear export. Nat Commun.

[70] Hohmann U (2024). A molecular switch orchestrates the nuclear export of human messenger RNA. BioRxiv.

[71] Xie Y (2023). Structural basis for high-order complex of SARNP and DDX39B to facilitate mRNP assembly. Cell Rep.

[72] Wilmes GM (2008). A genetic interaction map of RNA-processing factors reveals links between Sem1/Dss1-containing complexes and mRNA export and splicing. Mol Cell.

[73] Stewart M (2019). Structure and function of the TREX-2 complex. Subcell Biochem.

[74] Fischer T (2002). The mRNA export machinery requires the novel Sac3p-Thp1p complex to dock at the nucleoplasmic entrance of the nuclear pores. EMBO J.

[75] Wickramasinghe VO (2010). GANP enhances the efficiency of mRNA nuclear export in mammalian cells. Nucleus.

[76] Hautbergue GM (2008). Mutually exclusive interactions drive handover of mRNA from export adaptors to TAP. Proc Natl Acad Sci U S A.

[77] Nino CA (2013). mRNA nuclear export in yeast. Chem Rev.

[78] Aibara S (2015). The principal mRNA nuclear export factor NXF1:NXT1 forms a symmetric binding platform that facilitates export of retroviral CTE-RNA. Nucleic Acids Res.

[79] Paci G (2021). Cargo transport through the nuclear pore complex at a glance. J Cell Sci.

[80] Teplova M (2011). Structure-function studies of nucleocytoplasmic transport of retroviral genomic RNA by mRNA export factor TAP. Nat Struct Mol Biol.

[81] Aibara S (2015). Domain organization within the nuclear export factor Mex67:Mtr2 generates an extended mRNA binding surface. Nucleic Acids Res.

[82] Stewart M (2022). Function of the nuclear transport machinery in maintaining the distinctive compositions of the nucleus and cytoplasm. Int J Mol Sci.

[83] Li Z (2023). Nuclear export of pre-60S particles through the nuclear pore complex. Nature.

[84] Hodge CA (2011). The Dbp5 cycle at the nuclear pore complex during mRNA export I: dbp5 mutants with defects in RNA binding and ATP hydrolysis define key steps for Nup159 and Gle1. Genes Dev.

[85] Tran EJ (2007). The DEAD-box protein Dbp5 controls mRNA export by triggering specific RNA:protein remodeling events. Mol Cell.

[86] Strasser K (2000). Binding of the Mex67p/Mtr2p heterodimer to FXFG, GLFG, and FG repeat nucleoporins is essential for nuclear mRNA export. J Cell Biol.

[87] Strawn LA (2001). The GLFG regions of Nup116p and Nup100p serve as binding sites for both Kap95p and Mex67p at the nuclear pore complex. J Biol Chem.

[88] Adams RL (2017). Nup42 and IP6 coordinate Gle1 stimulation of Dbp5/DDX19B for mRNA export in yeast and human cells. Traffic.

[89] Adams RL (2014). Nucleoporin FG domains facilitate mRNP remodeling at the cytoplasmic face of the nuclear pore complex. Genetics.

[90] Adams RL, Wente SR (2020). Dbp5 associates with RNA-bound Mex67 and Nab2 and its localization at the nuclear pore complex is sufficient for mRNP export and cell viability. PLoS Genet.

[91] Alcazar-Roman AR (2006). Inositol hexakisphosphate and Gle1 activate the DEAD-box protein Dbp5 for nuclear mRNA export. Nat Cell Biol.

[92] Schmitt C (1999). Dbp5, a DEAD-box protein required for mRNA export, is recruited to the cytoplasmic fibrils of nuclear pore complex via a conserved interaction with CAN/Nup159p. EMBO J.

[93] Weirich CS (2004). The N-terminal domain of Nup159 forms a beta-propeller that functions in mRNA export by tethering the helicase Dbp5 to the nuclear pore. Mol Cell.

[94] Weirich CS (2006). Activation of the DExD/H-box protein Dbp5 by the nuclear-pore protein Gle1 and its coactivator InsP6 is required for mRNA export. Nat Cell Biol.

[95] Lin DH (2018). Structural and functional analysis of mRNA export regulation by the nuclear pore complex. Nat Commun.

[96] Peskin CS (1993). Cellular motions and thermal fluctuations: the Brownian ratchet. Biophys J.

[97] Stewart M (2007). Ratcheting mRNA out of the nucleus. Mol Cell.

[98] Montpetit B (2011). A conserved mechanism of DEAD-box ATPase activation by nucleoporins and InsP6 in mRNA export. Nature.

[99] Noble KN (2011). The Dbp5 cycle at the nuclear pore complex during mRNA export II: nucleotide cycling and mRNP remodeling by Dbp5 are controlled by Nup159 and Gle1. Genes Dev.

[100] Xie Y, Ren Y (2019). Mechanisms of nuclear mRNA export: a structural perspective. Traffic.

[101] von Moeller H (2009). The mRNA export protein DBP5 binds RNA and the cytoplasmic nucleoporin NUP214 in a mutually exclusive manner. Nat Struct Mol Biol.

[102] Fan JS (2009). Solution and crystal structures of mRNA exporter Dbp5p and its interaction with nucleotides. J Mol Biol.

[103] Hilbert M (2009). The mechanism of ATP-dependent RNA unwinding by DEAD box proteins. Biol Chem.

[104] Gray S (2022). The nucleoporin Gle1 activates DEAD-box protein 5 (Dbp5) by promoting ATP binding and accelerating rate limiting phosphate release. Nucleic Acids Res.

[105] Lin DH, Hoelz A (2019). The structure of the nuclear pore complex (an update). Annu Rev Biochem.

[106] Akey CW (2022). Comprehensive structure and functional adaptations of the yeast nuclear pore complex. Cell.

[107] Schultes EA (2005). Compact and ordered collapse of randomly generated RNA sequences. Nat Struct Mol Biol.

[108] Herschlag D (2018). The story of RNA folding, as told in epochs. Cold Spring Harb Perspect Biol.

[109] Corley M (2020). How RNA-binding proteins interact with RNA: molecules and mechanisms. Nat Rev Mol Cell Biol.

